# Supplementary material for: Two New Apotirucallane-Type Triterpenoids from the Pericarp of Toona sinensis and Their Ability to Reduce Oxidative Stress in Rat Glomerular Mesangial Cells Cultured under High-Glucose Conditions
Source: Molecules. 2020 Feb 12;25(4):801. doi: 10.3390/molecules25040801 (PMC7070558; doi:10.3390/molecules25040801)
Supplement: Supplementary file 1 [file molecules-25-00801-s001.pdf]

Supplementary data

Two new apotirucallane-type triterpenoids from the pericarp of *Toona sinesis* and their ability to reduce oxidative stress in rat glomerular mesangial cells cultured under high-glucose conditions

Di Liu, Rong-shen Wang, Lu-lu Xuan, Xiao-hong Wang *, and Wan-zhong Li *

School of Pharmacy, Weifang Medical University, Weifang 261053, Shandong Province, China

Abstract: Hyperglycemia is a strong risk factor for chronic complications of diabetes. Hyperglycemic conditions foster not only the production of reactive oxygen species (ROS), but also the consumption of antioxidants, leading to oxidative stress and promoting the occurrence and progression of complications. During our continuous search for antioxidant constituents from the pericarp of *Toona sinensis* (A. Juss.) Roem, we isolated two previously unreported apotirucallane-type triterpenoids, toonasinensin A (**1**) and toonasinensin B (**2**), together with five known apotirucallane-type triterpenoids (**3**–**7**) and two known cycloartane-type triterpenoids (**8**–**9**) from the pericarp. Compounds **8**–**9** were obtained from *T. sinensis* for the first time. Their structures were characterized based on interpretation of spectroscopic data (1D, 2D NMR, HR-ESI-MS) and comparison to previous reports. Compounds (**2**, **4**, **6**, **7**, and **9**) were able to inhibit proliferation against rat glomerular mesangial cells (GMCs) cultured under high glucose within a concentration of 80 μM. Compounds (**2**, **6**, and **7**) were tested for antioxidant activity attributable to superoxide dismutase (SOD), malondialdehyde (MDA), and ROS in *vitro*, and the results showed that compounds (**2**, **6**, and **7**) could significantly increase the level of SOD and reduce the level of MDA and

ROS. The current studies showed that apotirucallane-type triterpenoids (**2**, **6**, and **7**) might have the antioxidant effects against diabetic nephropathy.

Keywords: *Toona sinensis* (A. Juss.) Roem; apotirucallane-type triterpenoid; cycloartane-type triterpenoid; rat glomerular mesangial cells; oxidative stress

List of Figures

Figure S1: ^{1}H NMR spectrum of compound **1** in methanol-d_4 (500 MHz)

Figure S2: ^{13}C NMR spectrum of compound **1** in methanol-d_4 (125 MHz)

Figure S3: DEPT 135 spectrum of compound **1** in methanol-d_4

Figure S4: HMQC spectrum of compound **1** in methanol-d_4

Figure S5: HMBC spectrum of compound **1** in methanol-d_4

Figure S6: NOESY spectrum of compound **1** in methanol-d_4

Figure S7: HR-ESI-MS of compound **1**

Figure S8: ^{1}H NMR spectrum of compound **2** in methanol-d_4 (500 MHz)

Figure S9: ^{13}C NMR spectrum of compound **2** in methanol-d_4 (125 MHz)

Figure S10: DEPT 135 spectrum of compound **2** in methanol-d_4

Figure S11: HMQC spectrum of compound **2** in methanol-d_4

Figure S12: HMBC spectrum of compound **2** in methanol-d_4

Figure S13: NOESY spectrum of compound **2** in methanol-d_4

Figure S14: HR-ESI-MS of compound **2**

Figure S15: Cytotoxicity assay of compounds **1**–**9** in GMCs. GMCs were pretreated with compounds **1**–**9** (80 μM) and incubated for 48 h. GMCs proliferation was determined using the MTT assay. Values are expressed as mean ± SD of three independent experiments. $^{\#}P < 0.01$ relative to the 5.6 mM glucose (normal group, NG).

Figure S16: Extraction and partition of the pericarp of *Toona sinesis*

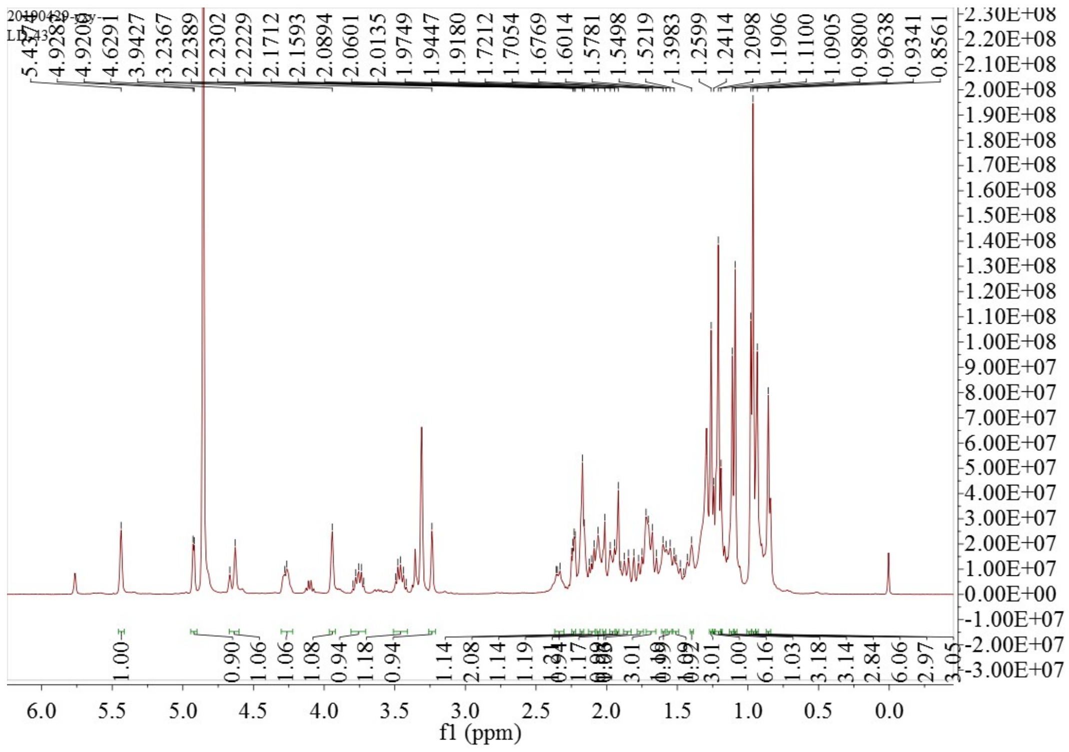

Figure S1: ^{1}H NMR spectrum of compound **1** in methanol-d_4 (500 MHz)

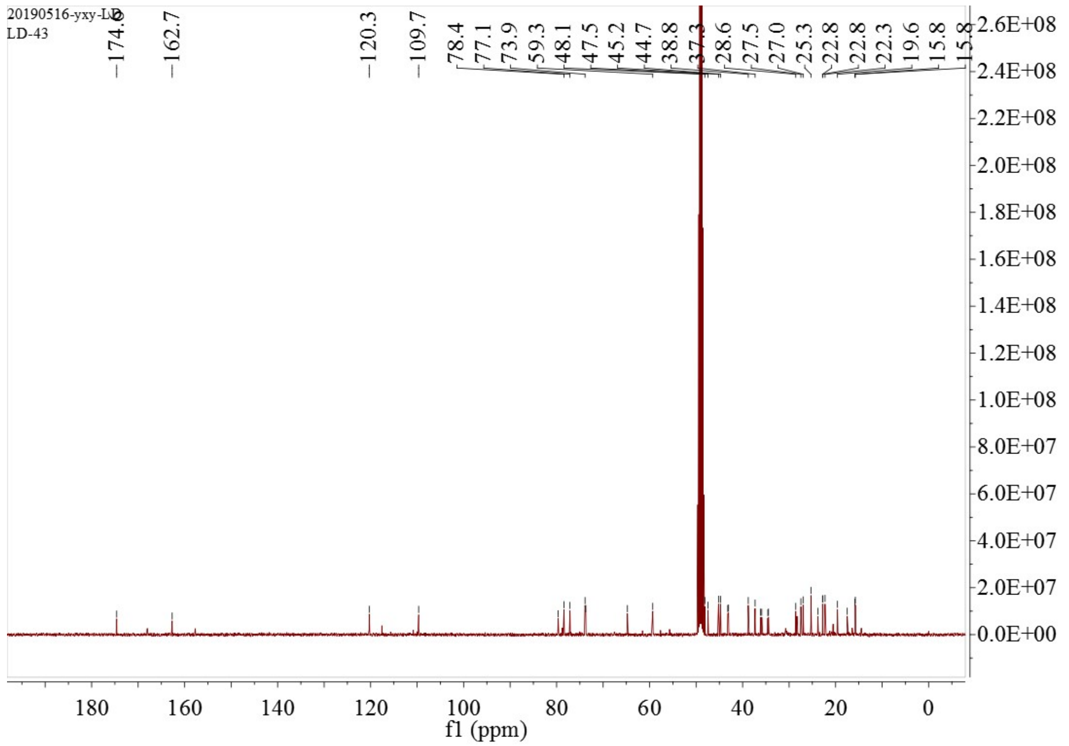

Figure S2: ^{13}C NMR spectrum of compound **1** in methanol-*d*$_4$ (125 MHz)

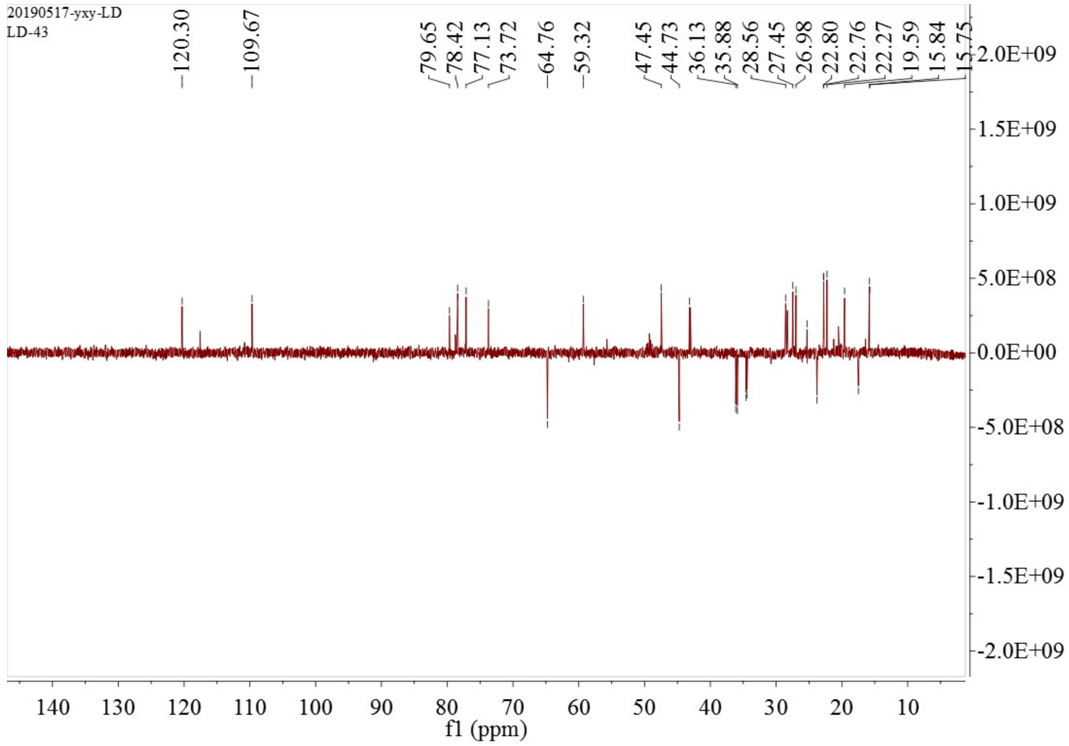

Figure S3: DEPT 135 spectrum of compound **1** in methanol-*d*$_4$

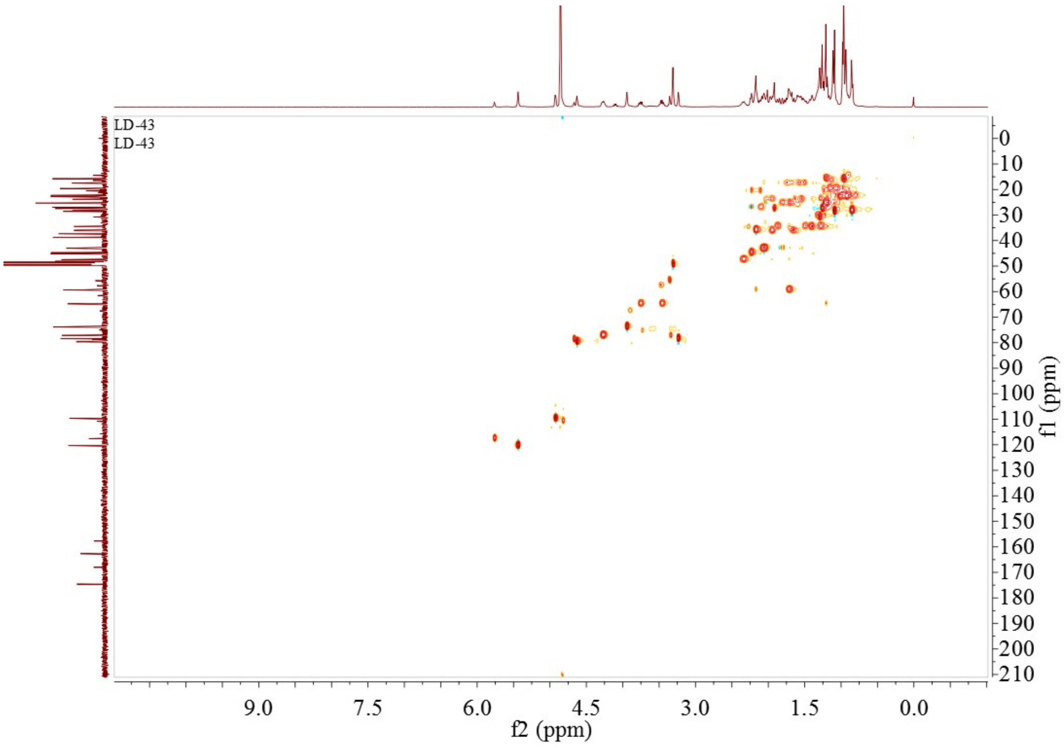

Figure S4: HMQC spectrum of compound **1** in methanol-d_4

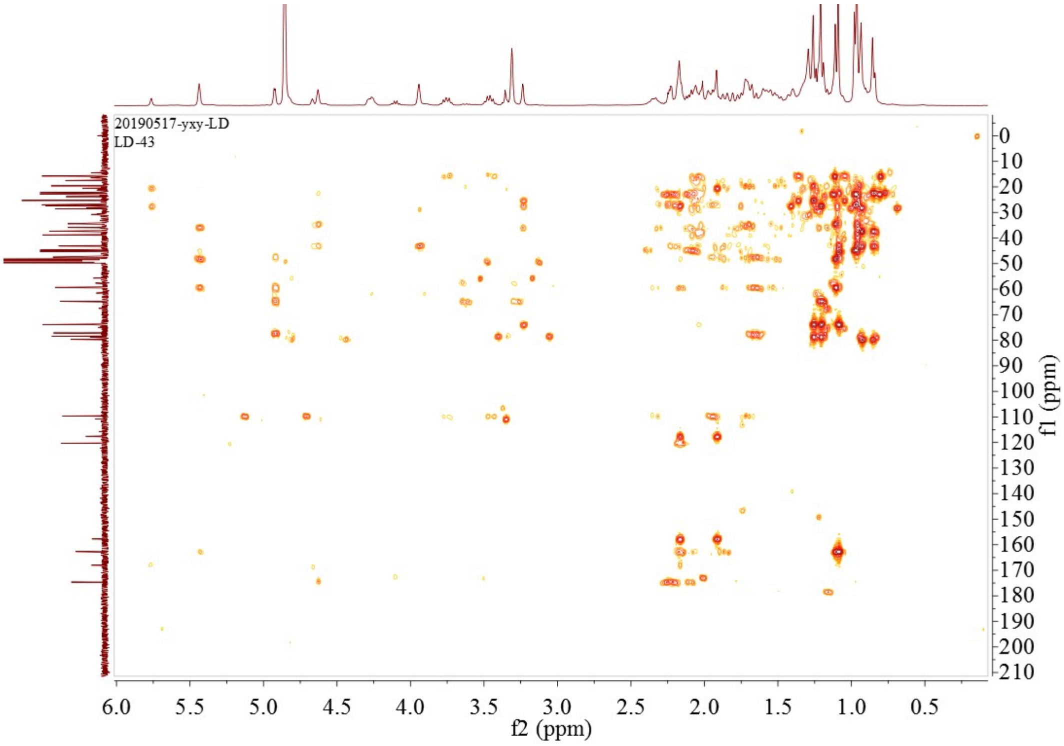

Figure S5: HMBC spectrum of compound **1** in methanol-d_4

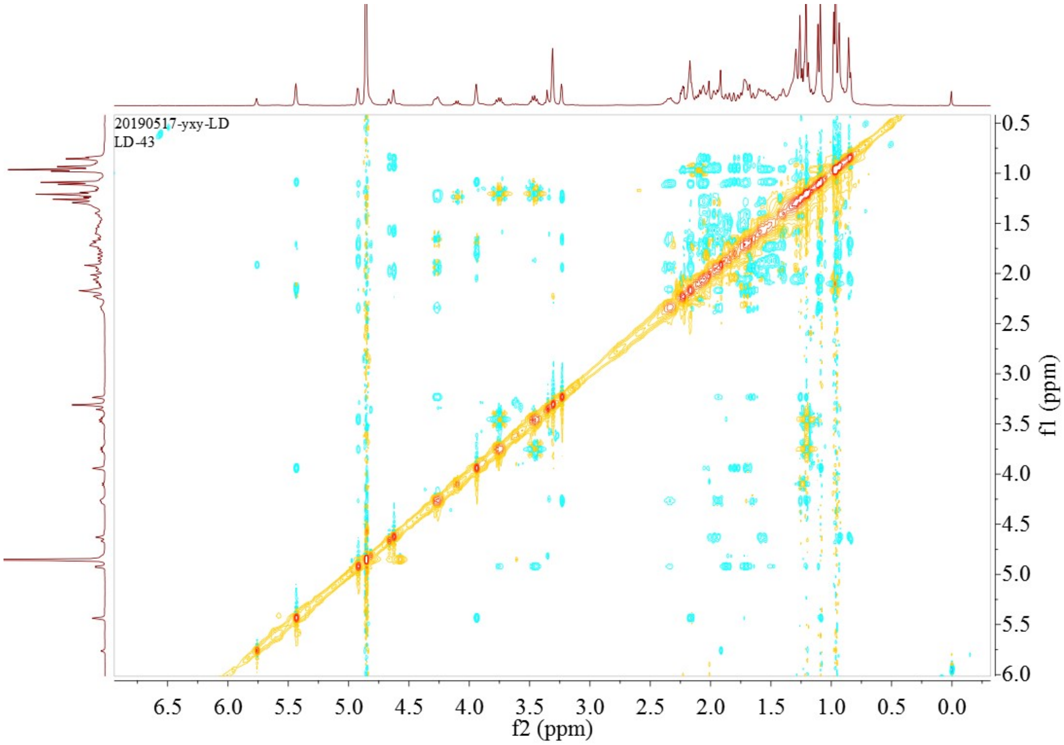

Figure S6: NOESY spectrum of compound **1** in methanol-d_4

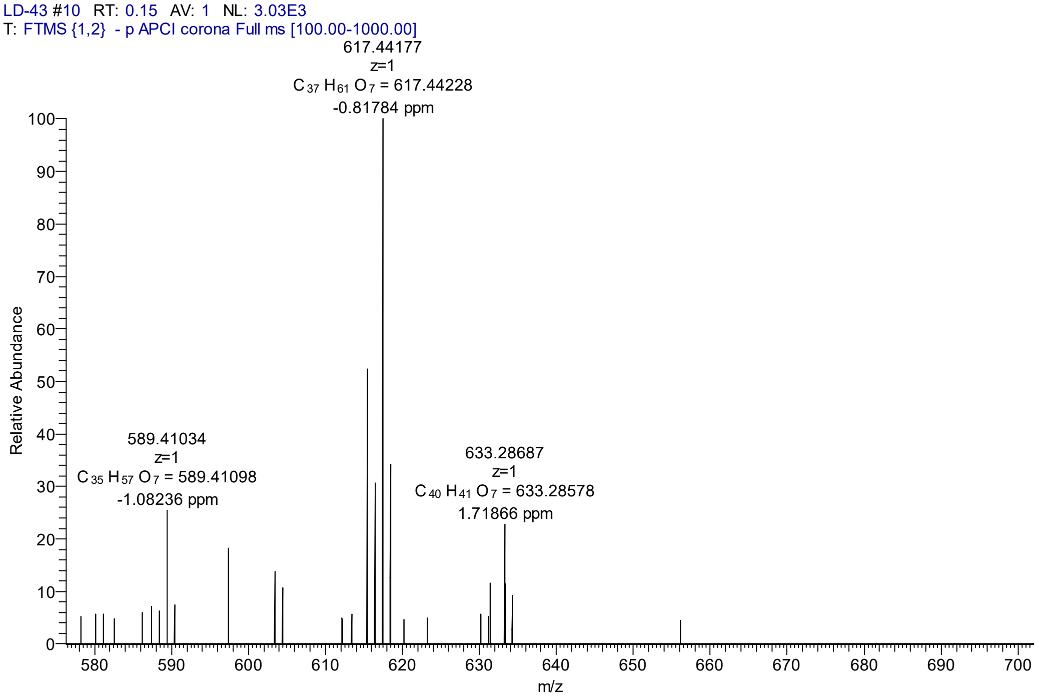

Figure S7: HR-ESI-MS of compound **1**

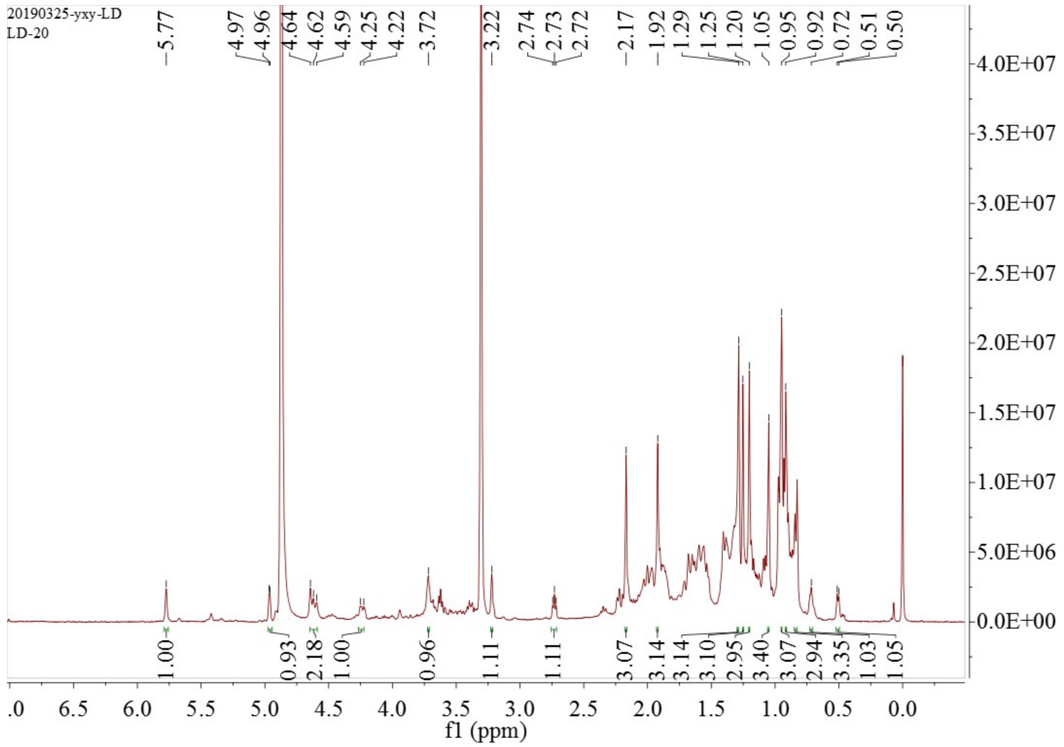

Figure S8: ^{1}H NMR spectrum of compound **2** in methanol-d_4 (500 MHz)

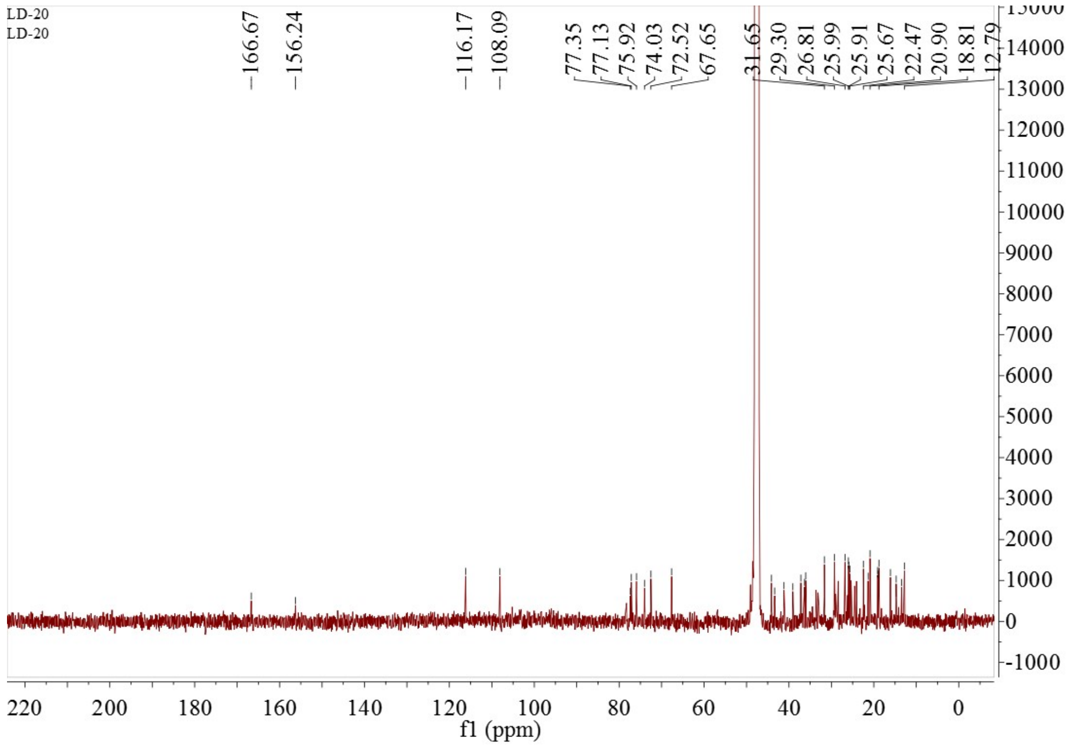

Figure S9: ^{13}C NMR spectrum of compound **2** in methanol-d_4 (125 MHz)

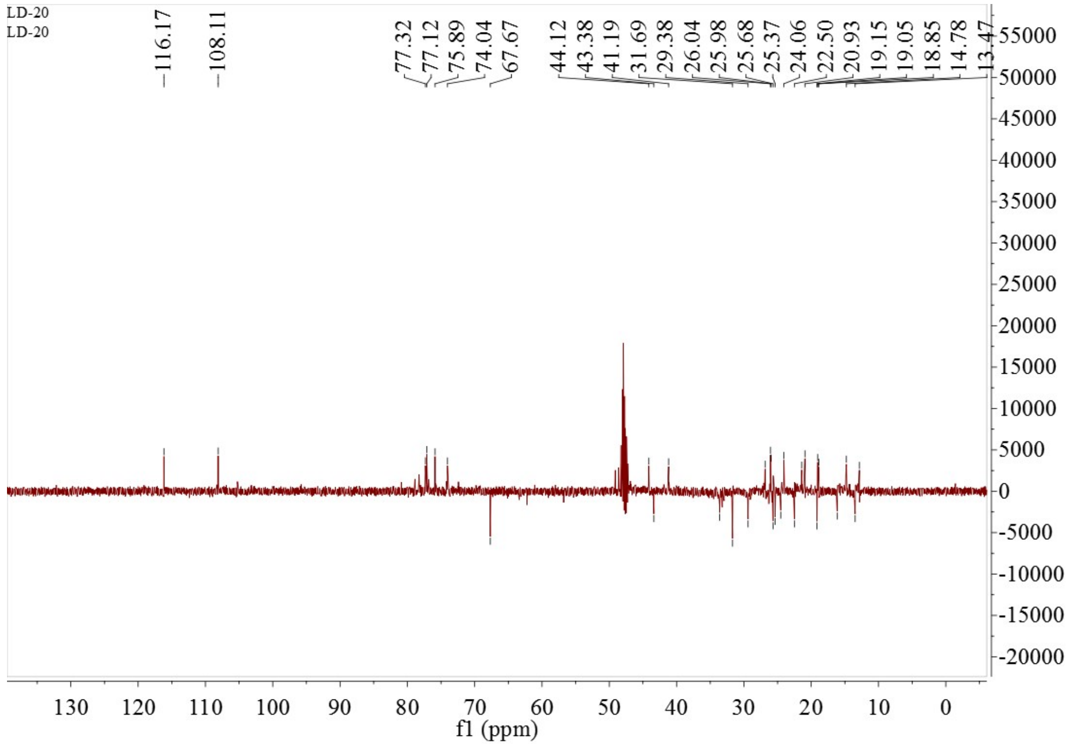

Figure S10: DEPT 135 spectrum of compound **2** in methanol-d_4

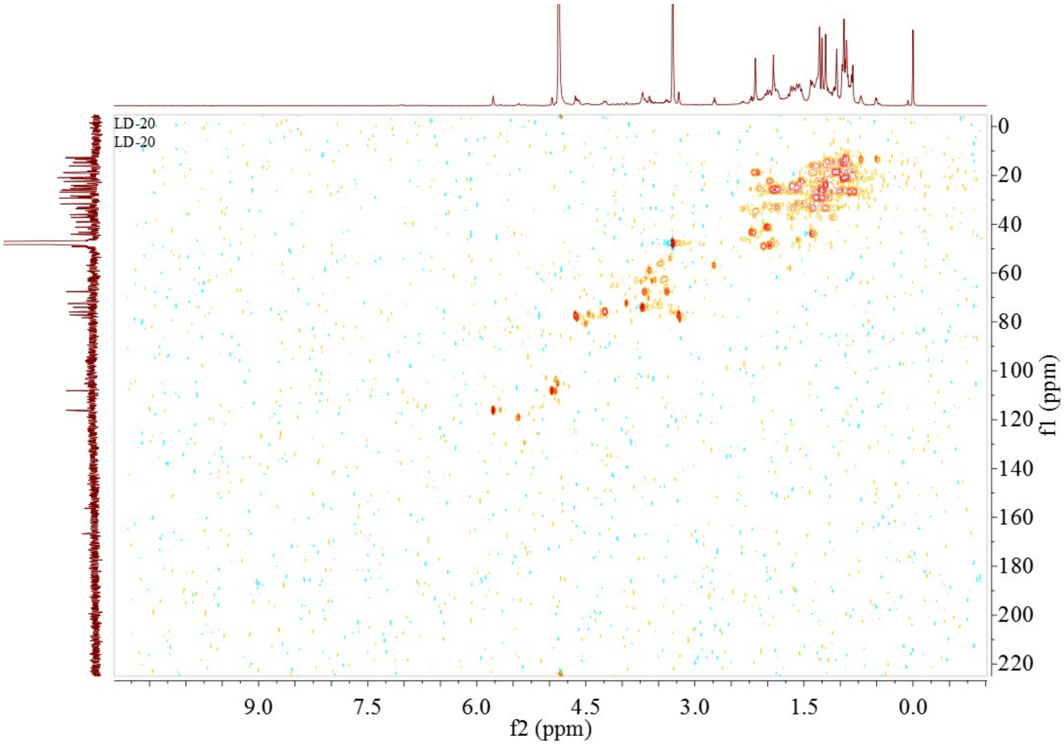

Figure S11: HMQC spectrum of compound **2** in methanol-d_4

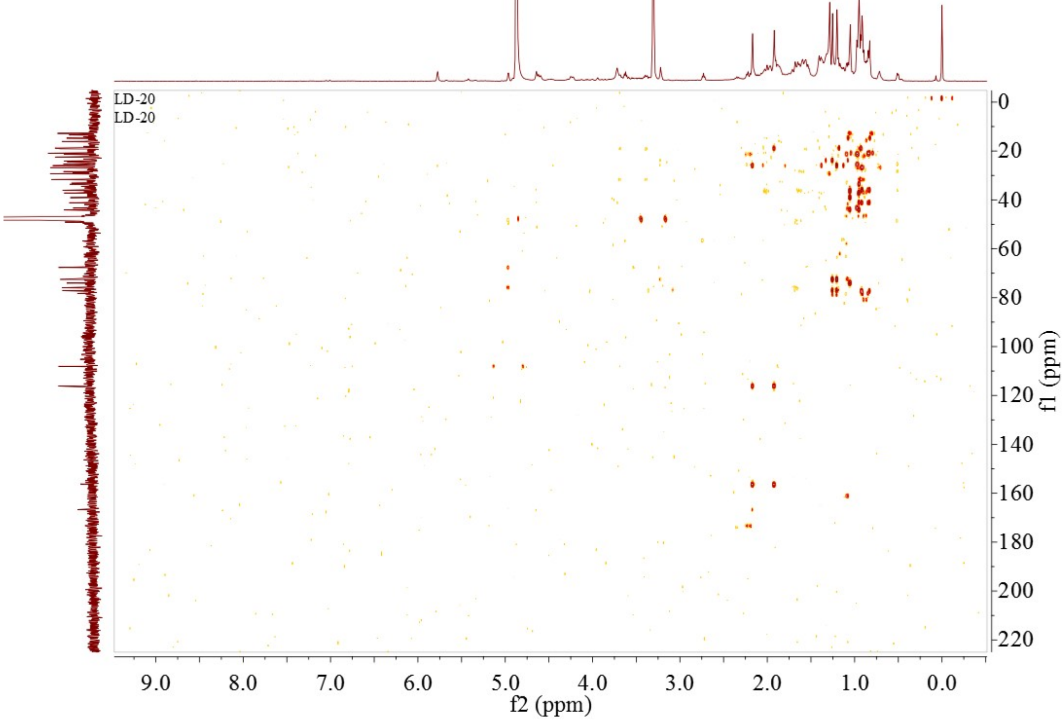

Figure S12: HMBC spectrum of compound **2** in methanol-d_4

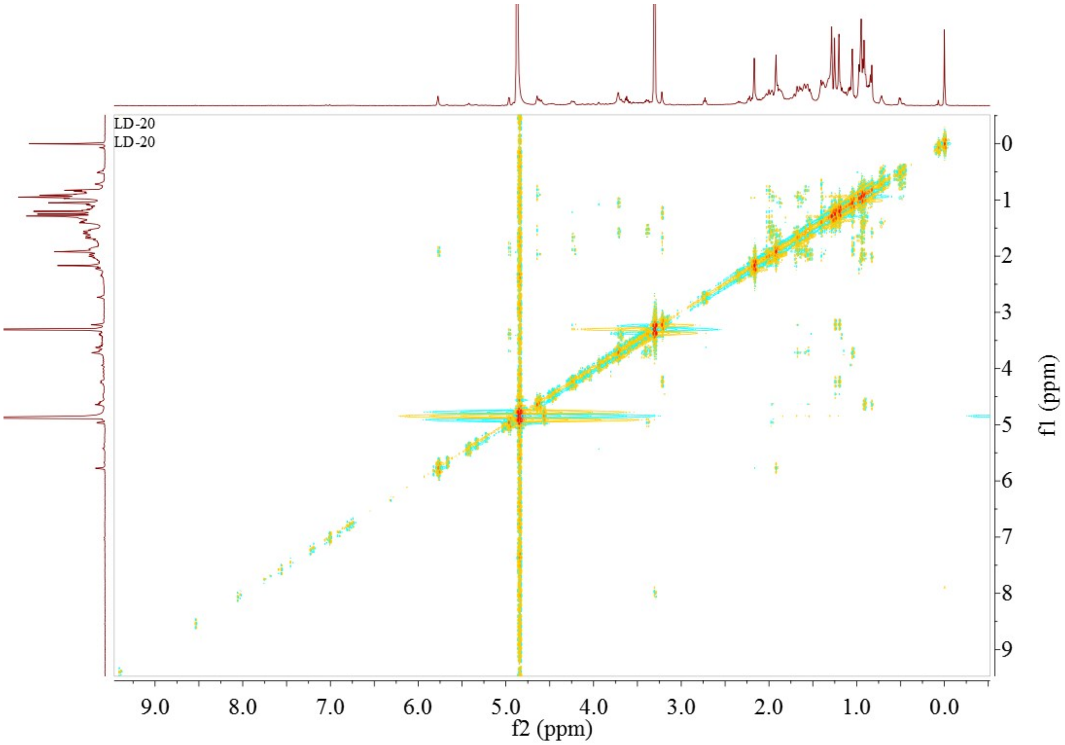

Figure S13: NOESY spectrum of compound **2** in methanol-d_4

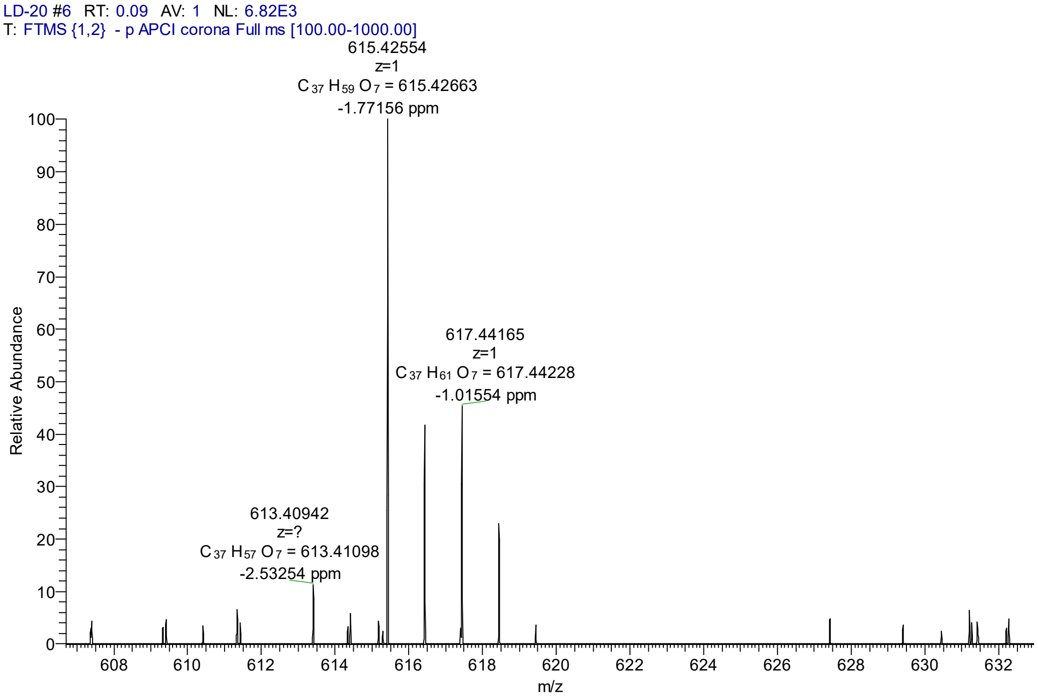

Figure S14: HR-ESI-MS of compound **2**

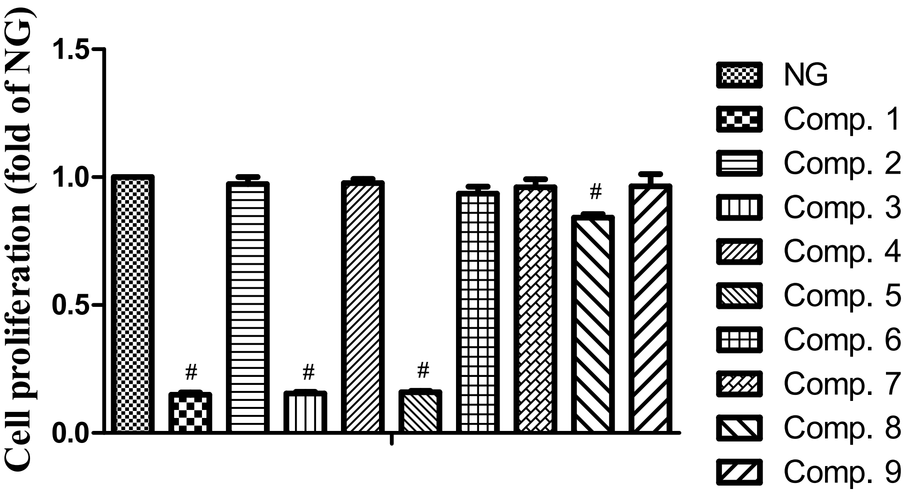

Figure S15: Cytotoxicity assay of compounds **1**–**9** in GMCs. GMCs were pretreated with compounds **1**–**9** (80 μM) and incubated for 48 h. GMCs proliferation was determined using the MTT assay. Values are expressed as mean ± SD of three independent experiments. #*P* < 0.01 relative to the 5.6 mM glucose (normal group, NG).

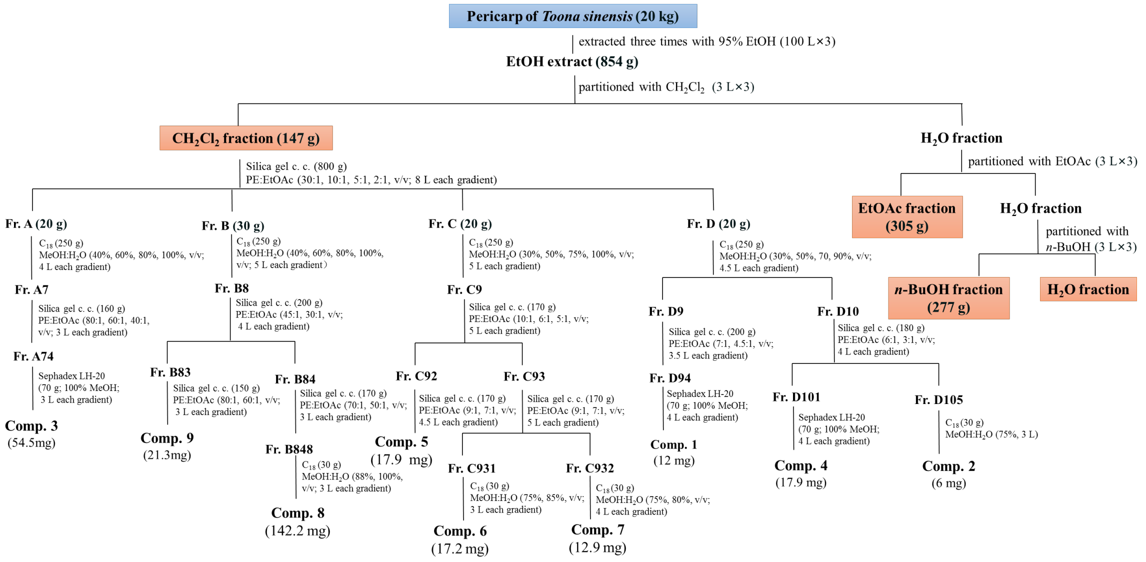

Figure S16: Extraction and partition of the pericarp of *Toona sinesis*
